# Circ_0051079 functions as an oncogenic regulator in osteosarcoma by leading to MAFB expression upregulation by competitively interacting with miR-1286

**DOI:** 10.1186/s13018-022-03297-w

**Published:** 2022-09-24

**Authors:** Zhong Huang, Pengcheng Chen, Ruipeng Jia, Yiheng Liu

**Affiliations:** Orthopedic Center, Haikou Affiliated Hospital of Central South University Xiangya School of Medicine, Haikou City, 570208 Hainan China

**Keywords:** circ_0051079, Osteosarcoma, miR-1286, MAFB

## Abstract

**Background:**

Circular RNAs are involved in various cellular processes of bone diseases by acting as miRNA sponges to regulate gene expression levels, including osteosarcoma (OS). This research concentrated on the molecular mechanism of circ_0051079 in OS progression.

**Methods:**

Reverse transcription-quantitative polymerase chain reaction assay was used for expression detection of circ_0051079, microRNA-1286 (miR-1286), and musculoaponeurotic fibrosarcoma oncogene homolog B (MAFB). Cell Counting Kit-8 assay and Edu assay were used for cell proliferation analysis. Cell apoptosis was evaluated using flow cytometry. Western blot was performed to measure protein levels. Migration and invasion were assessed via transwell assay. Interaction of circ_0051079/miR-1286 or miR-1286/MAFB was explored through a dual-luciferase reporter assay. In vivo research was carried out via tumor xenograft assay and immunohistochemistry staining.

**Results:**

Circ_0051079 expression was upregulated in OS. Downregulation of circ_0051079 reduced OS cell proliferation, migration, invasion, and accelerated apoptosis. Circ_0051079 interacted with miR-1286, and the tumor-inhibitory function of si-circ_0051079 was abolished by miR-1286 inhibition in OS cells. MAFB served as a target for miR-1286. OS cell progression was suppressed by miR-1286 overexpression via downregulating MAFB. Circ_0051079/miR-1286 resulted in expression change of MAFB in OS cells. Silencing circ_0051079 inhibited tumor growth in vivo via regulating the miR-1286/MAFB axis.

**Conclusion:**

The collective results elucidated that circ_0051079 contributed to OS progression via miR-1286-mediated upregulation of MAFB, confirming the interaction of circ_0051079/miR-1286/MAFB axis in OS.

**Supplementary Information:**

The online version contains supplementary material available at 10.1186/s13018-022-03297-w.

## Introduction

Osteosarcoma (OS) is the most common bone malignancy and usually occurred in adolescents with a rapid rate of bone growth [1]. The high metastatic tendency is a primary cause of treatment failure and poor prognosis, so it is imperative to investigate the molecular mechanisms of OS malignant progression [2]. Non-coding circular RNAs (circRNAs) and microRNAs (miRNAs) have been indicated to play vital impacts in the physiopathologic processes of OS [3]. The regulatory networks associated with circRNAs and miRNAs may contribute to understanding the pathogenesis of OS.

CircRNAs are single-stranded and closed-loop RNAs derived from specific back-splicing, which play a role as oncogenic or inhibiting regulators in the development of various cancers [4, 5], containing OS [6]. CircRNAs can serve as miRNA sponges to induce gene regulation at post-transcriptional levels, further affecting cancer initiation and development, including OS [7]. For instance, circ_0008259 might increase PDCD4 level via competitively binding to miR-21-5p, thus delaying cell proliferation and metastatic potential in OS [8]. In addition, Zhang et al. elucidated that circ_0051079 facilitated OS proliferation and metastasis through mediating the miR-26a-5p/TGF-β1 axis [9]. The other molecular network for oncogenic circ_0051079 remains to be researched.

MiRNAs are small RNA transcripts that play an essential role in regulating the behavior of multiple tumor cells via interacting with the 3′untranslated regions (3′UTRs) of target mRNAs [10, 11], including OS cells [12]. MicroRNA-1286 (miR-1286) is aberrantly downregulated in OS patients and acts as an inhibitory role in OS cells by targeting RAB13 [13]. Musculoaponeurotic fibrosarcoma oncogene homolog B (MAFB) is a key oncogene for tumorigenesis in OS [14]. The target relationship between miR-1286 and MAFB is fully unknown. Moreover, whether circ_0051079 can target miR-1286 to modulate MAFB expression is still to be investigated.

It is assumed that circ_0051079 is a potential miR-1286 sponge and that MAFB is the downstream gene of miR-1286. The regulatory mechanism of circ_0051079 with miR-1286 and MAFB is a key point of this study, aiming to discover different miRNA/mRNA axis supporting the function of circ_0051079 in OS. Our findings discovered that circ_0051079 might be a potential therapeutic target for OS.

## Materials and methods

### Human tissues

Sixty OS patients at Haikou Affiliated Hospital of Central South University Xiangya School of Medicine have signed informed consent for this research. All participants underwent diagnostic core needle biopsy using a disposable sterile biopsy instrument (Trauson Medical Instrument Corporation, China). None of the patients received radiotherapy or chemotherapy before surgery. OS tissues (*n* = 60) and normal adjacent tissues (*n* = 60) were collected after patients were treated with surgical resection, then samples were placed instantly into liquid nitrogen and preserved at − 80 °C. Morphologically normal muscle tissues that were more than 5 cm from the cancerous tissues were used as adjacent noncancerous tissues. Patients with other medical treatments before surgery were excluded from the current study. Declaration of Helsinki was strictly followed during the operating processes, and sample collection was approved by the Ethics Committee of Haikou Affiliated Hospital of Central South University Xiangya School of Medicine. The detailed clinical characteristics of patients are described in Table 1.

**Table 1:** Correlation between clinicopathologic parameters of osteosarcoma patients and circ_0051079 expression

Parameter	Case	Circ_0051079 expression		*P* value
		Low (*n* = 30)	High (*n* = 30)	
Age (years)				0.197
≤ 60	31	13	18	
> 60	29	17	12	
Gender				0.432
Female	35	16	19	
Male	25	14	11	
Tumor size				0.029*
≤ 5 cm	20	14	6	
> 5 cm	40	16	24	
TNM stages				0.0001*
I–II	25	20	5	
III	35	10	25	

*TNM* tumor-node-metastasis

*P < 0.05

### Cell culture and transfection

SaoS-2 and U2OS cell lines (COBIOER, Nanjing, China) were purchased for OS research, and hFOB1.19 (COBIOER) was used as a negative control cell line. Dulbecco’s modified eagle medium (DMEM; Gibco, Carlsbad, CA, USA) was replenished with 10% fetal bovine serum (FBS; Gibco) and 1% antibiotic solution (Gibco). Cell culture with a mixed medium was performed in a 5% CO_2_ incubator at 37 °C, while hFOB1.19 cells were grown at 33.5 °C. Lipofectamine™ 3000 Kit (Invitrogen, Carlsbad, CA, USA) was applied for transfection of SaoS-2 and U2OS cells. Small interfering RNA targeting circ_0051079 or negative control (si-circ_0051079: 5′-AGTCATCATTGCCAAGACTGT-3′, si-NC: 5′-AATTCTCCGAACGTGTCACGT-3′), mimic for miR-1286 or control (miR-1286: 5′-TGCAGGACCAAGATGAGCCCT-3′, miR-NC: 5′-TTCTCCGAACGTGTCACGTTT-3′), inhibitor for miR-1286 or control (anti-miR-1286: 5′-AGGGCTCATCTTGGTCCTGCA-3′, anti-miR-NC: 5′-CAGTACTTTTGTGTAGTACAA-3′) were obtained from GenePharma (Shanghai, China). MAFB sequence was cloned into pcDNA (Invitrogen) to generate pcDNA-MAFB (MAFB) vector. The above oligonucleotides or vectors were diluted with Opti-MEM® Reduced Serum Medium, then added with Lipofectamine™ 3000 reagent, and reacted for 15 min. Cell incubation with transfection complexes was performed for 48 h, followed by further analysis.

### Reverse transcription-quantitative polymerase chain reaction (RT-qPCR) assay

Human tissues and cells were lysed with TransZol reagent (TransGen, Beijing, China), followed by DNase treatment to eliminate DNA contamination. Then, total RNA was reversely transcribed to complementary DNA (cDNA) via *EasyScript*® All-in-One First-Strand cDNA Synthesis SuperMix for qPCR (TransGen). *TransStart*® Green qPCR SuperMix (TransGen) was used for the preparation of the quantification system and specific primers (Table 2), followed by expression calculation through the 2^−∆∆Ct^ method [15]. Total RNA was digested using 5 U/μg RNase R (GENESEED, Guangzhou, China) at 37 °C, then circ_0051079 and GAPDH were quantified by RT-qPCR. Nuclear and cytoplasmic RNAs were isolated by PARIS™ Kit (Invitrogen), then circ_0051079 localization was analyzed using RT-qPCR. Beta-actin (β-actin) served as a reference gene for circ_0051079 and mRNA levels. U6 was used to normalize the expression of miR-1286.

**Table 2:** Primer sequences used for RT-qPCR

Name	Primers for PCR (5′–3′)	
hsa_circ_0051079	Forward	CCGCTACTACGCCATGAAGA
	Reverse	GGTACGCTGTCACCTAGCTC
MAFB	Forward	AGAGAGAACCGAGAGGTCCC
	Reverse	AGCAGAGGGGAGGATCTGTT
miR-1286	Forward	GTATGAGTGCAGGACCAAGATG
	Reverse	CTCAACTGGTGTCGTGGAG
GAPDH	Forward	GACAGTCAGCCGCATCTTCT
	Reverse	GCGCCCAATACGACCAAATC
U6	Forward	CTCGCTTCGGCAGCACA
	Reverse	AACGCTTCACGAATTTGCGT

### Cell Counting Kit-8 (CCK-8) assay

2 × 10^3^ SaoS-2 and U2OS cells were incubated with 10 μL/well CCK-8 solution (Sigma, St. Louis, MO, USA) at various time points of transfection. After incubation for 4 h, a microplate reader (Bio-Rad, Hercules, CA, USA) was employed for detecting the optical density value at 450 nm.

### Edu assay

Proliferation examination was carried out by Edu cell proliferation kit (Beyotime, Shanghai, China), according to user manuals. 4 × 10^4^ OS cells were incubated with Edu solution and the nucleus was stained with diamidine phenylindole (DAPI). Then, cell analysis was carried out under a fluorescence microscope (Olympus, Tokyo, Japan). Edu^+^ cells were defined as SaoS-2 and U2OS cells with simultaneous staining of EdU and DAPI.

### Flow cytometry

After transfection for 72 h, 5 × 10^4^ OS cells were applied for apoptosis determination via Annexin V Apoptosis Kit (BD Biosciences, San Diego, CA, USA). 10 µL Annexin V-fluorescein isothiocyanate (Annexin V-FITC) and 5 µL propidium iodide (PI) were pipetted to cells and incubated at room temperature for 20 min. Subsequently, cells were determined via the flow cytometer (BD Biosciences) and the apoptosis rate was calculated by a percentage of FITC^+^/PI^−^ or FITC^+^/PI^+^ stained cells in total cells.

### Western blot

Total proteins from cultured cells and collected tissues were extracted using Radioimmunoprecipitation assay buffer (Beyotime) containing protease inhibitor (cocktail, Roche, Basel, Switzerland). The sample concentration was examined through BCA Protein Assay Kit (Beyotime), and then, western blot analysis was implemented as previously depicted [16]. In short, 50 μg of each protein sample was separated by 10% SDS-PAGE and then transferred to a nitrocellulose membrane (Millipore, Bedford, MA, USA). After being blocked with 5% skim milk at room temperature for 1 h, the membrane was incubated with the primary antibody against B-cell lymphoma-2 (Bcl-2; ab32124), Bcl-2 associated X (Bax; ab32503), E-cadherin (ab40772), Vimentin (ab92547), N-cadherin (ab18203), MAFB (ab65953), and β-actin (ab8227) from Abcam (Cambridge, UK) with 1:1000 dilution at 4 °C overnight. Incubation of Goat anti-rabbit IgG H&L (HRP) secondary antibody (Abcam, ab205718) at 1:5000 was performed for 45 min. Electrochemiluminescence (ECL) Substrate Kit (Abcam) was used for the exhibition of protein blots, followed by expression analysis in Image J software (NIH, Bethesda, MD, USA).

### Transwell assay

Transwell chamber (Corning Inc., Corning, NY, USA) was exploited for the assessment of cell migration and invasion abilities. 1 × 10^4^ SaoS-2 and U2OS cells were resuspended in serum-free medium, then seeded in the transwell upper chamber for migration examination. In addition, the upper chamber was coated with matrigel (Corning Inc.) and inoculated with 1 × 10^5^ cells for invasion analysis. DMEM medium containing 10% FBS was added into the lower chamber. After 24 h of incubation, cells passed through the membranes and were dyed with 0.1% crystal violet (Sigma). Migrated or invaded cells under five visual fields were counted, and pictures were taken at 100× magnification through an inverted microscope (Olympus).

### Dual-luciferase reporter assay

Binding sites were predicted by Starbase3.0 (http://starbase.sysu.edu.cn). Wild-type (WT) luciferase reporter plasmids of circ_0051079 and MAFB 3′UTR were constructed through molecular cloning into pmirGLO (Promega, Madison, WI, USA). The positive plasmids circ_0051079-WT and MAFB 3′UTR-WT contained binding sites of miR-1286. Meanwhile, mutant-type (MUT) plasmids circ_0051079-MUT WT and MAFB 3′UTR-MUT containing mutated sites of miR-1286 were used as negative controls. SaoS-2 and U2OS cells were co-transfected with miR-1286 or miR-NC and WT or MUT plasmid. After 48 h of transfection, the cells were collected and the luciferase activity was detected using the Dual-Luciferase Reporter Kit (Promega).

### Xenograft tumor assay

First of all, Lentiviral vectors expressing short hairpin RNA against circ_0051079 (sh-circ_0051079) or negative control (sh-NC) from GeneChem (Shanghai, China) were infected into SaoS-2 cells in media containing 8 μg/ml of polybrene, followed by selection using puromycin. BALB/c nude mice (Vital River Laboratory Animal Technology Co., Ltd., Beijing, China) were subcutaneously injected with 2 × 10^6^ transfected cells. There were 6 mice in sh-NC or sh-circ_0051079 group. Tumor volume (Length × Width^2^ × 0.5) was measured weekly. 35 d later, mice were euthanatized by indrawing the flow rate of CO_2_ and tumors were weighed. RT-qPCR or western blot was performed for expression detection of circ_0051079, miR-1286 and MAFB in tumor tissues. Detection of Ki67 protein level (Abcam, ab15580, 1:100) was implemented using Immunohistochemistry (IHC) assay [17]. This assay was ratified by the Animal Ethical Committee of Haikou Affiliated Hospital of Central South University Xiangya School of Medicine.

### Statistical analysis

Assays were repeated three times and three paralleled samples, then data of the mean ± standard deviation were analyzed by SPSS 22.0 (SPSS Inc., Chicago, IL, USA). Pearson’s correlation coefficient was carried out to analyze linear relations in OS tissues. Then, statistical difference was assessed through Student’s *t*-test and analysis of variance (ANOVA) followed by Tukey’s test. *P* < 0.05 showed a significant difference, statistically.

## Results

### Circ_0051079 was highly expressed in OS

**Fig. 1:**
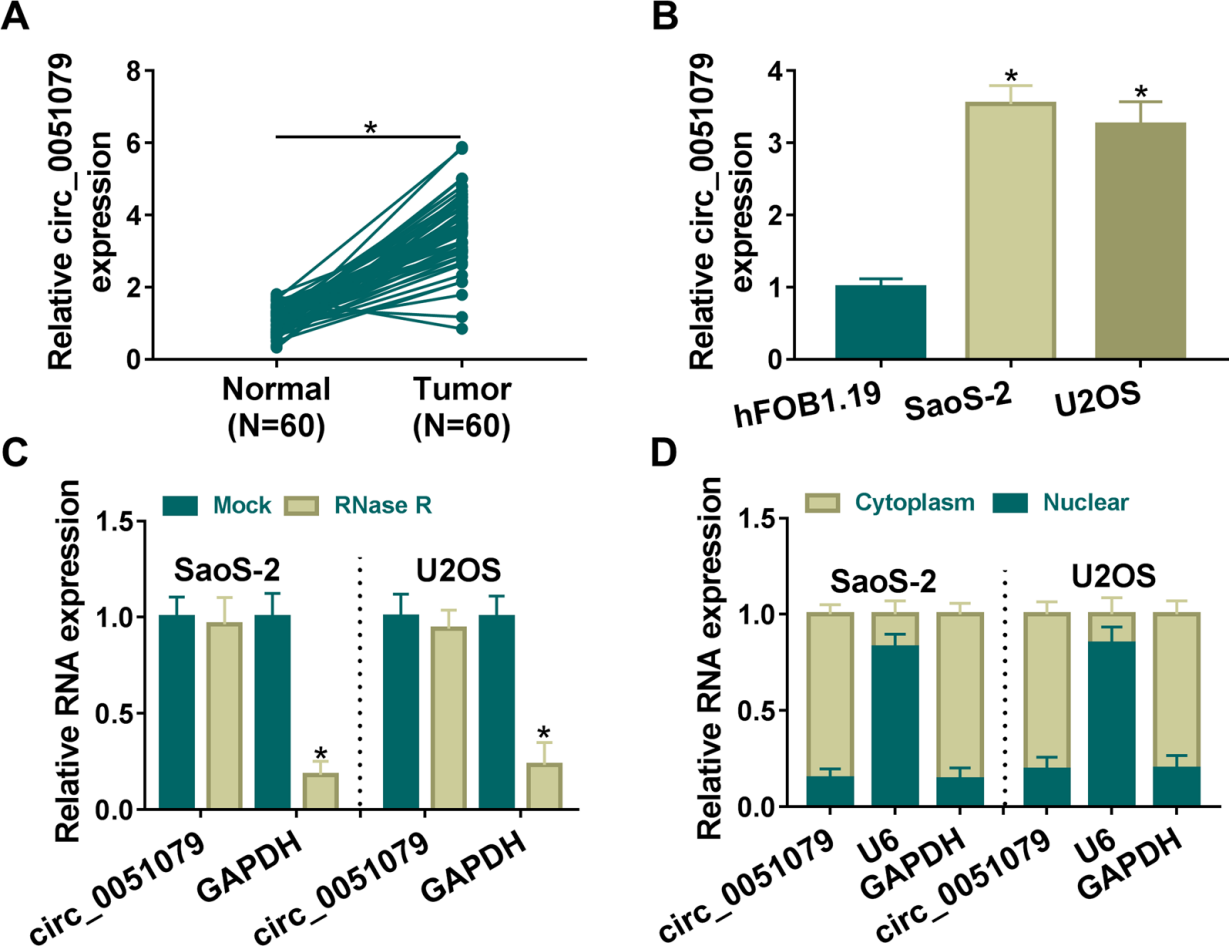
Circ_0051079 was highly expressed in OS. **A**, **B** RT-qPCR was performed for circ_0051079 quantification in OS/normal tissues (**A**) and SaoS-2, U2OS or hFOB1.19 cells (**B**). **C** Circ_0051079 and GAPDH stabilities were assessed by RNase R digestion and RT-qPCR detection. **D** Circ_0051079, U6 and GAPDH levels in cytoplasm and nucleus of SaoS-2 and U2OS cells were measured through RT-qPCR. *P < 0.05

First, we investigated the expression level of circ_0051079 in 60 OS patients. As shown in Fig. 1A, the circ_0051079 level was markedly increased in tumor samples contrasted with normal samples. According to the median value of circ_0051079 expression, OS patients were divided into the high circ_0051079 expression group and the circ_0051079 low expression. Then, the Kaplan–Meier survival curves demonstrated that OS patients in low circ_0051079 level group had a higher survival rate than those in the high circ_0051079 level group (Additional file 1: Fig. S1A). Meanwhile, circ_0051079 expression was associated with tumor size and TNM stages (Table 1). Also, circ_0051079 was significantly upregulated in SaoS-2 and U2OS cells relative to hFOB1.19 cells (Fig. 1B). Stability analysis showed that circ_0051079 was more resistant to RNase R digestion than linear GAPDH in SaoS-2 and U2OS cells (Fig. 1C). Localization assay demonstrated that circ_0051079 was mainly localized in the cytoplasm of SaoS-2 and U2OS cells, with nuclear U6 and cytoplasmic GAPDH as control groups (Fig. 1D). High stability and cell localization identified that circ_0051079 was an upregulated circRNA.

### OS cell proliferation, migration, and invasion were inhibited but apoptosis was promoted after the silencing of circ_0051079

**Fig. 2:**
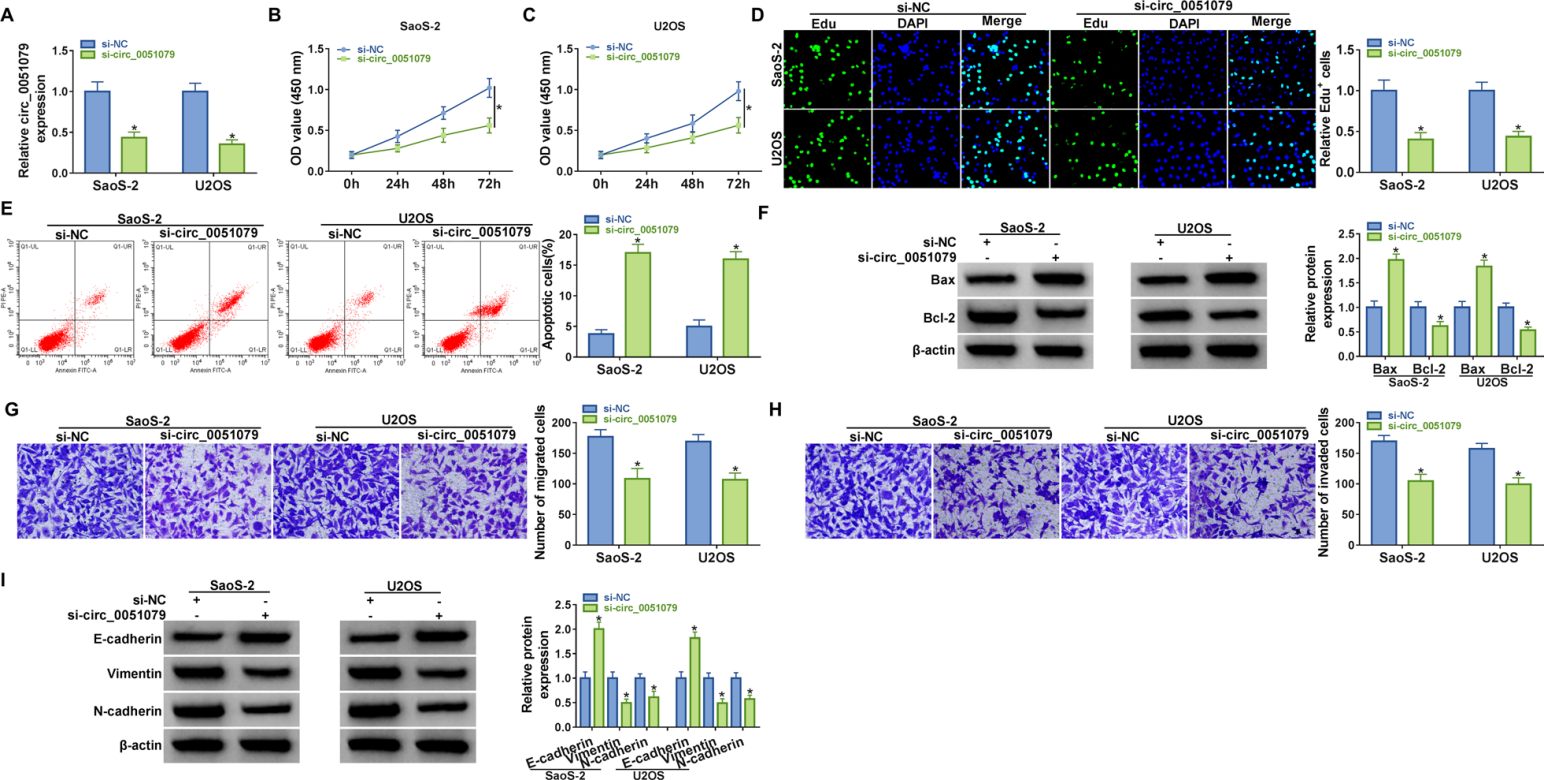
OS cell proliferation, migration, and invasion were inhibited but apoptosis was promoted after the silence of circ_0051079. Si-NC and si-circ_0051079 were transfected into SaoS-2 and U2OS cells, respectively. **A** Level detection of circ_0051079 was carried out by RT-qPCR. **B**–**D** Measurement of cell proliferation was taken via CCK-8 assay (**B**–**C**) and Edu assay (**D**). **E** Examination of apoptosis rate was conducted using flow cytometry. **F** Protein detection of apoptotic markers was implemented via western blot. **G**–**H** Assessment of migration (**G**) and invasion (H) was performed by transwell assay. **I** Determination of EMT-related proteins was conducted through western blot. *P < 0.05

Circ_0051079 was knocked down by specific siRNA, and the transfection efficiency of si-circ_0051079 was excellent in SaoS-2 and U2OS cells (Fig. 2A). CCK-8 assay showed that cell growth was delayed in the si-circ_0051079 group compared to the si-NC group (Fig. 2B, C). Also, Edu assay exhibited that cell proliferation was suppressed in si-circ_0051079-transfected SaoS-2 and U2OS cells relative to si-NC-transfected cells (Fig. 2D). Besides, knockdown of circ_0051079 induced SaoS-2 and U2OS cell apoptotic rate (Fig. 2E), as proved by increased pro-apoptotic Bax and decreased anti-apoptotic Bcl-2 (Fig. 2F). Transwell assay should that migrated and invaded cells were reduced after downregulation of circ_0051079 (Fig. 2G–H). Furthermore, EMT-related protein markers were examined using western blot. Silencing circ_0051079 elevated E-cadherin protein expression but inhibited Vimentin and N-cadherin protein levels in SaoS-2 and U2OS cells (Fig. 2I). In short, circ_0051079 aggravated cell malignant behaviors of OS.

### Circ_0051079 exhibited sponge function of miR-1286

**Fig. 3:**
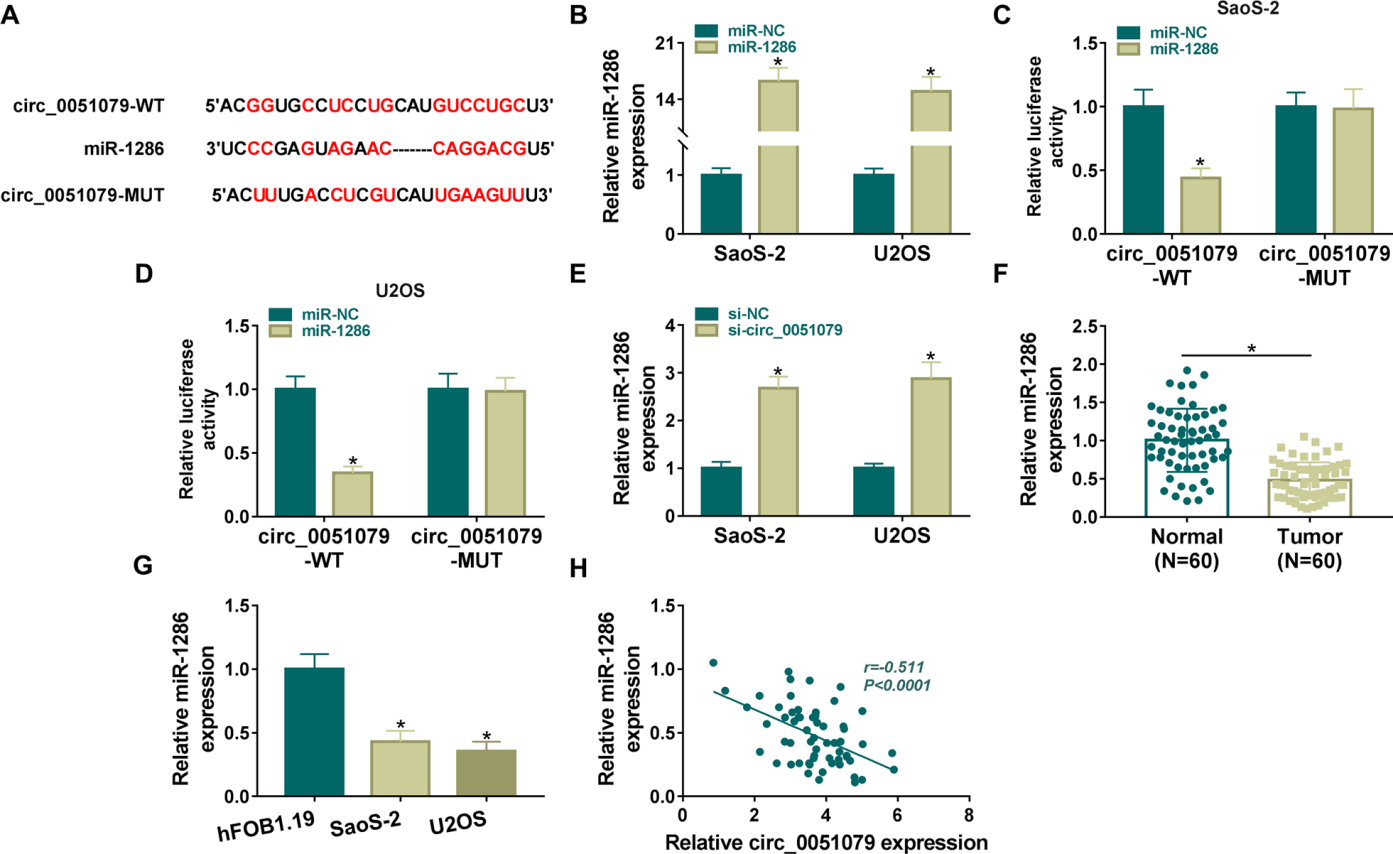
Circ_0051079 exhibited the sponge function of miR-1286. **A** Starbase3.0 was used for bioinformatics analysis between circ_0051079 and miR-1286 sequences. **B** The miR-1286 level was examined using RT-qPCR in SaoS-2 and U2OS cells transfected with miR-NC or miR-1286. **C**, **D** Circ_0051079 and miR-1286 target binding analysis was performed using dual-luciferase reporter assay. **E** RT-qPCR was applied to measure miR-1286 expression after transfection with si-NC or si-circ_0051079. **F**, **G** Expression quantification of miR-1286 was conducted via RT-qPCR in OS samples (**F**) and SaoS-2/U2OS cells (**G**). **H** Linear association between circ_0051079 and miR-1286 was analyzed through Pearson’s correlation coefficient. *P < 0.05

By performing target prediction in Starbase3.0, circ_0051079 was noticed to contain multiple binding sites with miR-1286 sequence (Fig. 3A). RT-qPCR data displayed that miR-1286 transfection resulted in a significant enhancement in miR-1286 in SaoS-2 and U2OS cells relative to miR-NC transfection (Fig. 3B). With co-transfection of miR-1286 and circ_0051079-WT (rather than circ_0051079-MUT), luciferase activity was found to be suppressed in SaoS-2 and U2OS cells (Fig. 3C, D). The miR-1286 expression was obviously enhanced by si-circ_0051079 compared with si-NC group (Fig. 3E). In addition, miR-1286 was aberrantly downregulated in OS tissues (Fig. 3F) and SaoS-2/U2OS cells (Fig. 3G) contrasted to normal tissues and hFOB1.19 cells. Based on the median value of miR-1286 level, 60 OS patients were classified into miR-1286 high and low groups. Using Kaplan–Meier analysis, we determined the positive association between miR-1286 level and the overall survival rate of OS patients (Additional file 1: Fig. S1B). A negative relation (*r* = −0.511, *P* < 0.0001) was detected between levels of circ_0051079 and miR-1286 in OS samples (Fig. 3H). Circ_0051079 directly interacted with miR-1286 in OS.

### Inhibition of miR-1286 counterbalanced anti-tumor response of si-circ_0051079 in OS cell progression

**Fig. 4:**
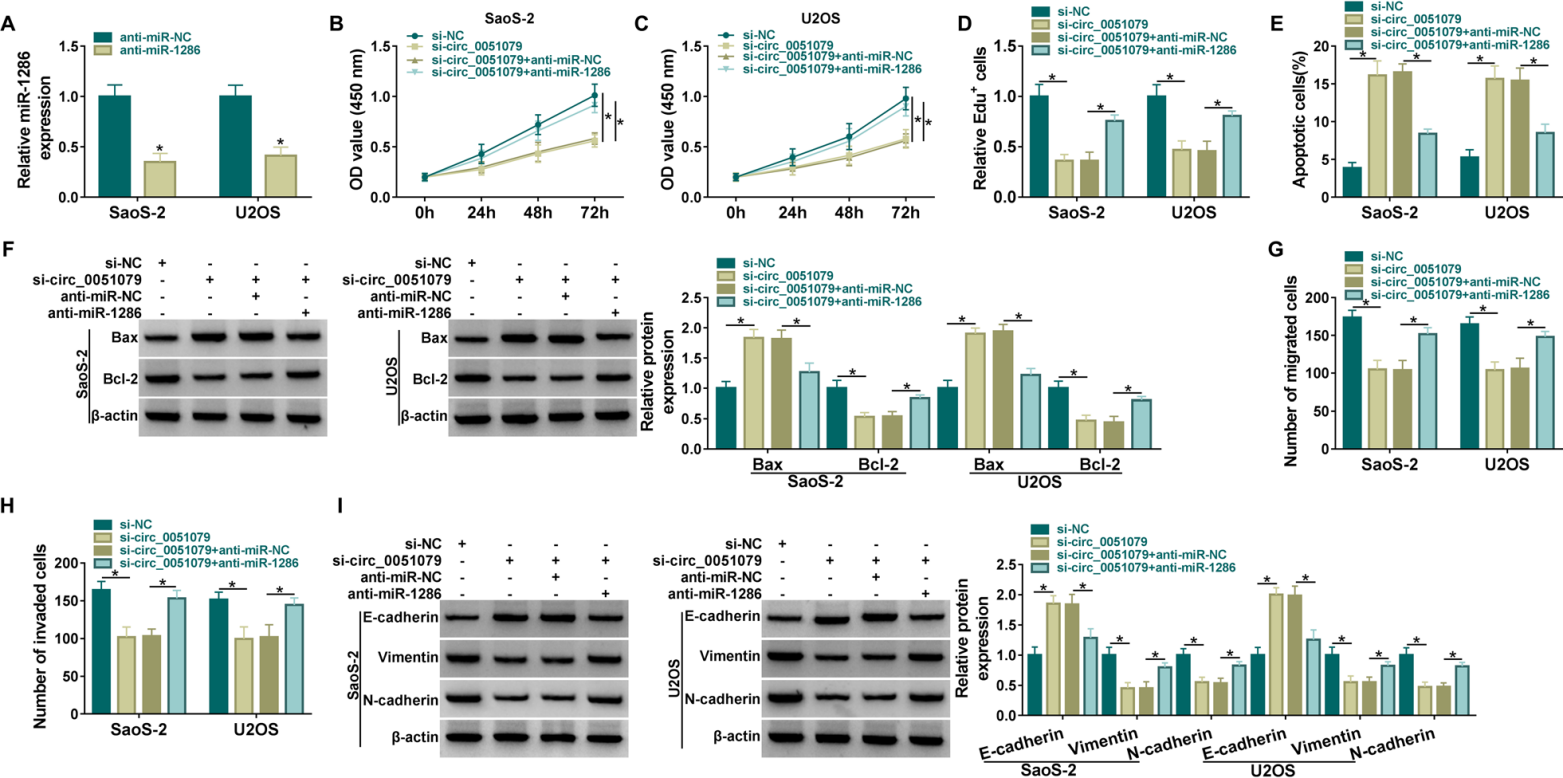
Inhibition of miR-1286 counterbalanced anti-tumor response of si-circ_0051079 in OS cell progression. **A** Inhibition efficacy of anti-miR-1286 was evaluated via RT-qPCR assay. **B**–**I** SaoS-2 and U2OS cells were performed with si-NC, si-circ_0051079, si-circ_0051079 + anti-miR-NC, si-circ_0051079 + anti-miR-1286 transfection. **B**–**D** Cell proliferation was analyzed through CCK-8 assay (**B**, **C**) and Edu assay (**D**). **E** Apoptosis rate was examined using flow cytometry. **F** Apoptotic markers were determined by western blot. **G**, **H** Migrated (**G**) and invaded (**H**) cells were detected via transwell assay. **I** EMT-associated proteins were measured using western blot. *P < 0.05

RT-qPCR analysis demonstrated that miR-1286 level was evidently reduced after anti-miR-1286 transfection in SaoS-2 and U2OS cells compared with cells transfected with anti-miR-NC (Fig. 4A). OD value (Fig. 4B, C) and Edu^+^ cells (Fig. 4D) were increased in si-circ_0051079 + anti-miR-1286 group by contrast to si-circ_0051079 + anti-miR-NC group. Flow cytometry (Fig. 4E) demonstrated that miR-1286 inhibitor reversed si-circ_0051079-mediated acceleration of cell apoptosis, as proved by decreased Bax and increased Bcl-2 (Fig. 4F). Additionally, migrated and invaded cell number reduction caused by si-circ_0051079 was offset by anti-miR-1286 in SaoS-2 and U2OS cells (Fig. 4G, H). Protein level changes of E-cadherin, Vimentin, and N-cadherin were also relieved after the downregulation of miR-1286 (Fig. 4I). Thus, circ_0051079 regulated OS cell progression partly via targeting miR-1286.

### MAFB was a downstream target of miR-1286

**Fig. 5:**
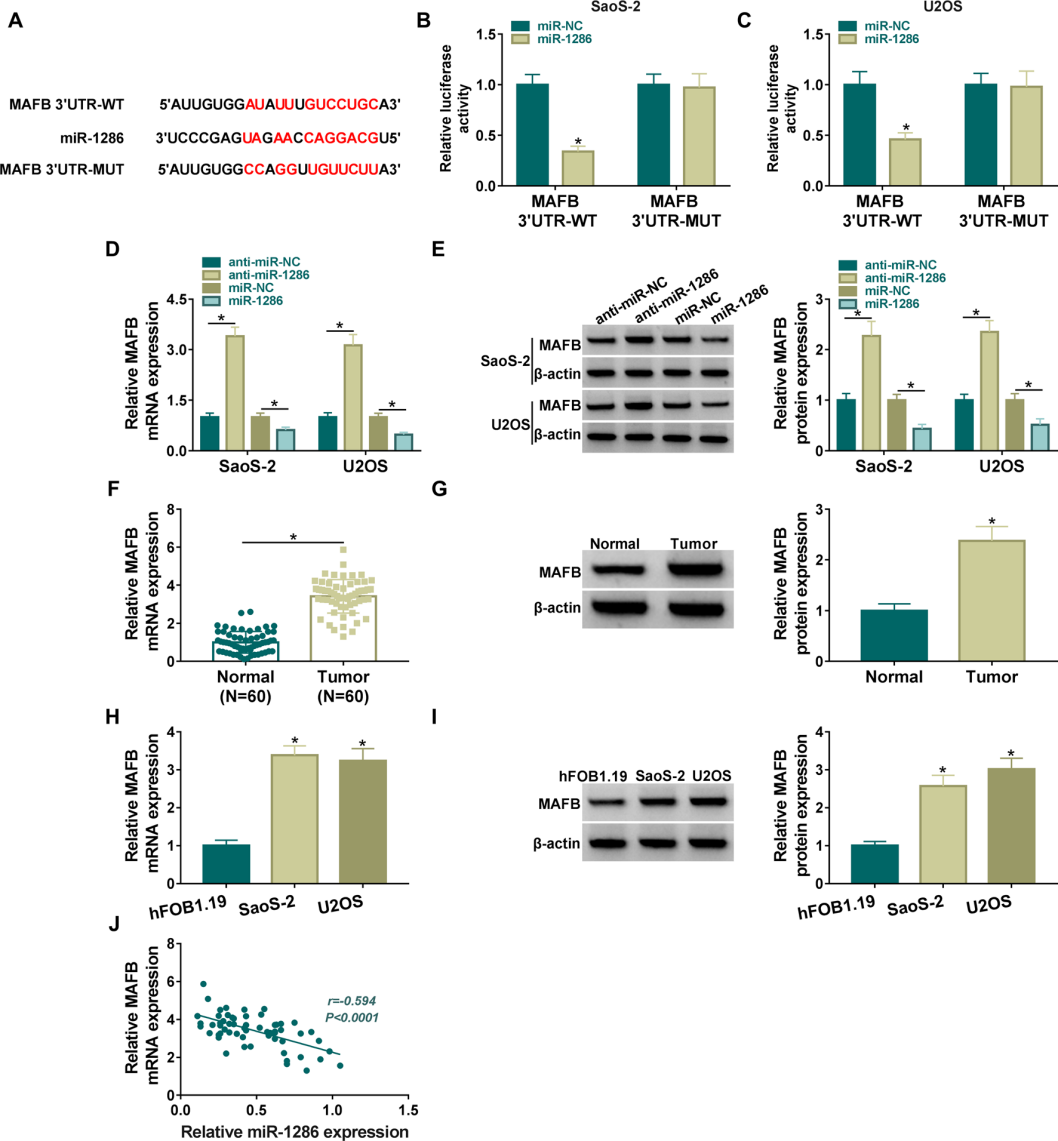
MAFB was a downstream target of miR-1286. **A** Binding sites between MAFB 3′UTR and miR-1286 sequences. **B**, **C** Dual-luciferase reporter assay was conducted to validate interaction between miR-1286 and MAFB. **D**, **E** MAFB mRNA and protein levels were detected by RT-qPCR and western blot after transfection of anti-miR-1286, miR-2186 and corresponding control groups. F–**I** RT-qPCR and western blot were applied for expression analysis of MAFB in OS tissues (**F**, **G**) and SaoS-2/U2OS cells (**H**, **I**). **J** Pearson’s correlation coefficient was performed for linear analysis between MAFB and miR-1286 in OS samples. *P < 0.05

Starbase3.0 predicted multiple binding sites between MAFB 3′UTR and miR-1286 sequences (Fig. 5A). Afterword, a dual-luciferase reporter assay exhibited that luciferase activity of WT-MAFB 3′UTR group, rather than MUT-MAFB 3′UTR group, was inhibited by overexpression of miR-1286 in SaoS-2 and U2OS cells (Fig. 5B, C). Hence, miR-1286 could directly bind to MAFB 3′UTR in OS cells. In addition, MAFB mRNA and protein expression levels were increased by miR-1286 overexpression and were decreased by miR-1286 downregulation in SaoS-2 and U2OS cells (Fig. 5D, E). Compared with normal samples and hFOB1.19 cells, mRNA and protein levels of MAFB were significantly upregulated in OS samples (Fig. 5F, G) and SaoS-2/U2OS cells (Fig. 5H, I). Besides, we found that OS patients with higher expression of MAFB possessed lower survival time (Additional file 1: Fig. S1C). Pearson’s correlation coefficient indicated that miR-1286 expression was negatively associated with MAFB expression (*r* = −0.594, *P* < 0.0001) in OS tissues (Fig. 5J). Taken together, miR-1286 targeted MAFB in OS.

### MiR-1286 functioned as a tumor repressor in OS cells by reducing MAFB expression

**Fig. 6:**
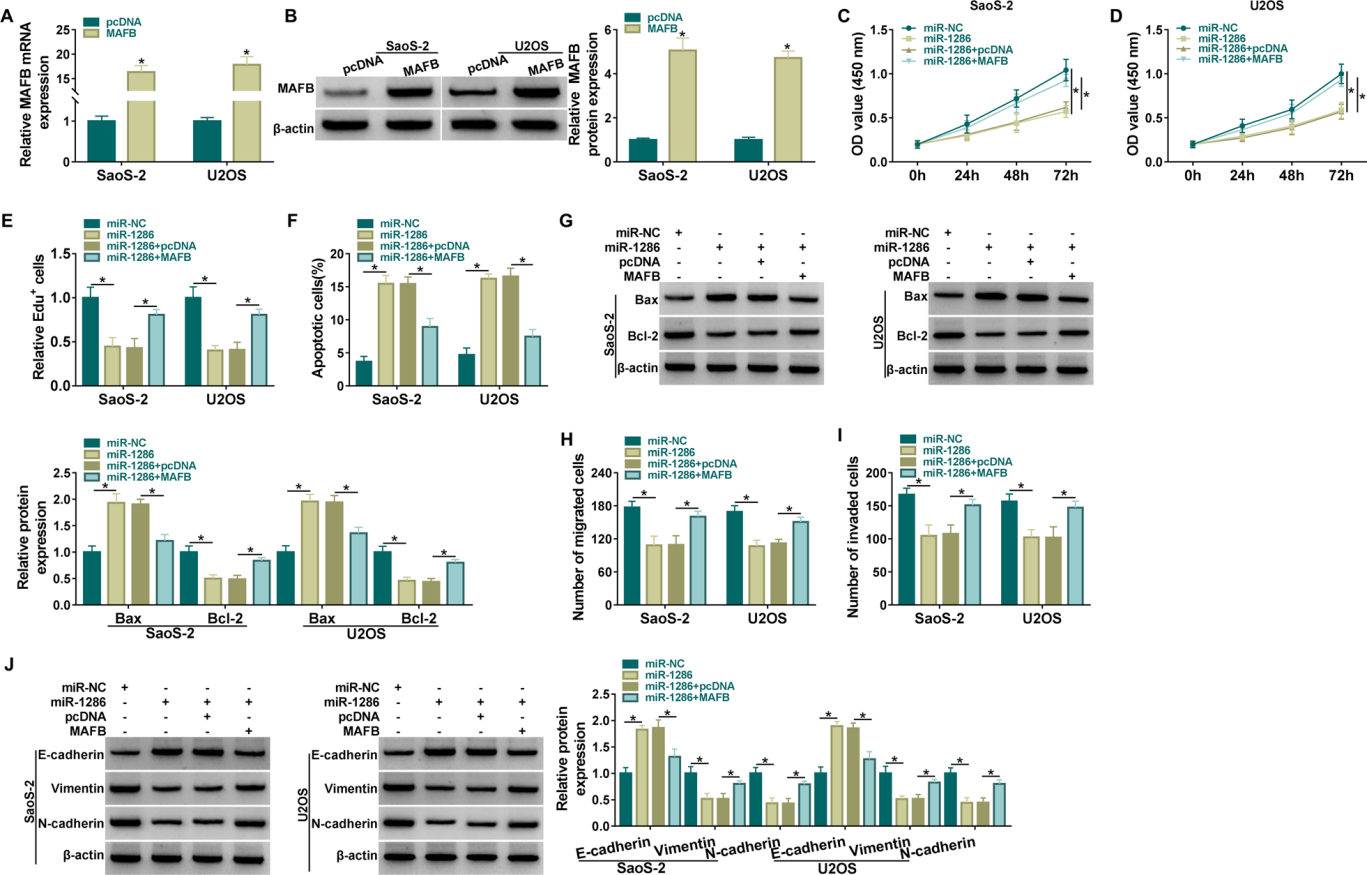
MiR-1286 functioned as a tumor repressor in OS cells by reducing MAFB expression. **A**, **B** RT-qPCR and western blot were used for MAFB quantification in pcDNA or MAFB transfection group. **C**–**J** SaoS-2 and U2OS cells were transfected with miR-1286, miR-1286 + MAFB or matched controls. **C**–**E** CCK-8 assay (**C**, **D**) and Edu assay (**E**) were used to determine cell proliferation. **F** Flow cytometry was used for examination of apoptosis rate. **G** Western blot was applied to detect levels of apoptotic proteins. **H**, **I** Transwell assay was applied for analysis of cell migration (**H**) and invasion (**I**). **J** Western blot was exploited for protein detection of EMT-associated markers. *P < 0.05

MAFB overexpression was achieved by constructing an expression vector. RT-qPCR and western blot results manifested that the mRNA and protein expression of MAFB in SaoS-2 and U2OS cells were significantly upregulated after the transfection of pcDNA-MAFB (Fig. 6A, B). CCK-8 (Fig. 6C, D) and Edu assay (Fig. 6E) revealed that miR-1286 overexpression suppressed cell proliferation ability, which was subsequently restored by MAFB upregulation. Apart from that, MAFB introduction also attenuated miR-1286-induced promotion of apoptosis rate (Fig. 6F) and Bax upregulation or Bcl-2 downregulation (Fig. 6G) in SaoS-2 and U2OS cells. The inhibitory effects of miR-1286 on migrated and invaded cells were attenuated by MAFB upregulation (Fig. 6H, I). Meanwhile, overexpression of MAFB notably counteracted miR-1286-caused E-cadherin protein elevation and Vimentin or N-cadherin protein inhibition (Fig. 6J). Overall, miR-1286 inhibited OS cell development through downregulating MAFB level.

### Circ_0051079 interacted with miR-1286 to change the MAFB expression

**Fig. 7:**
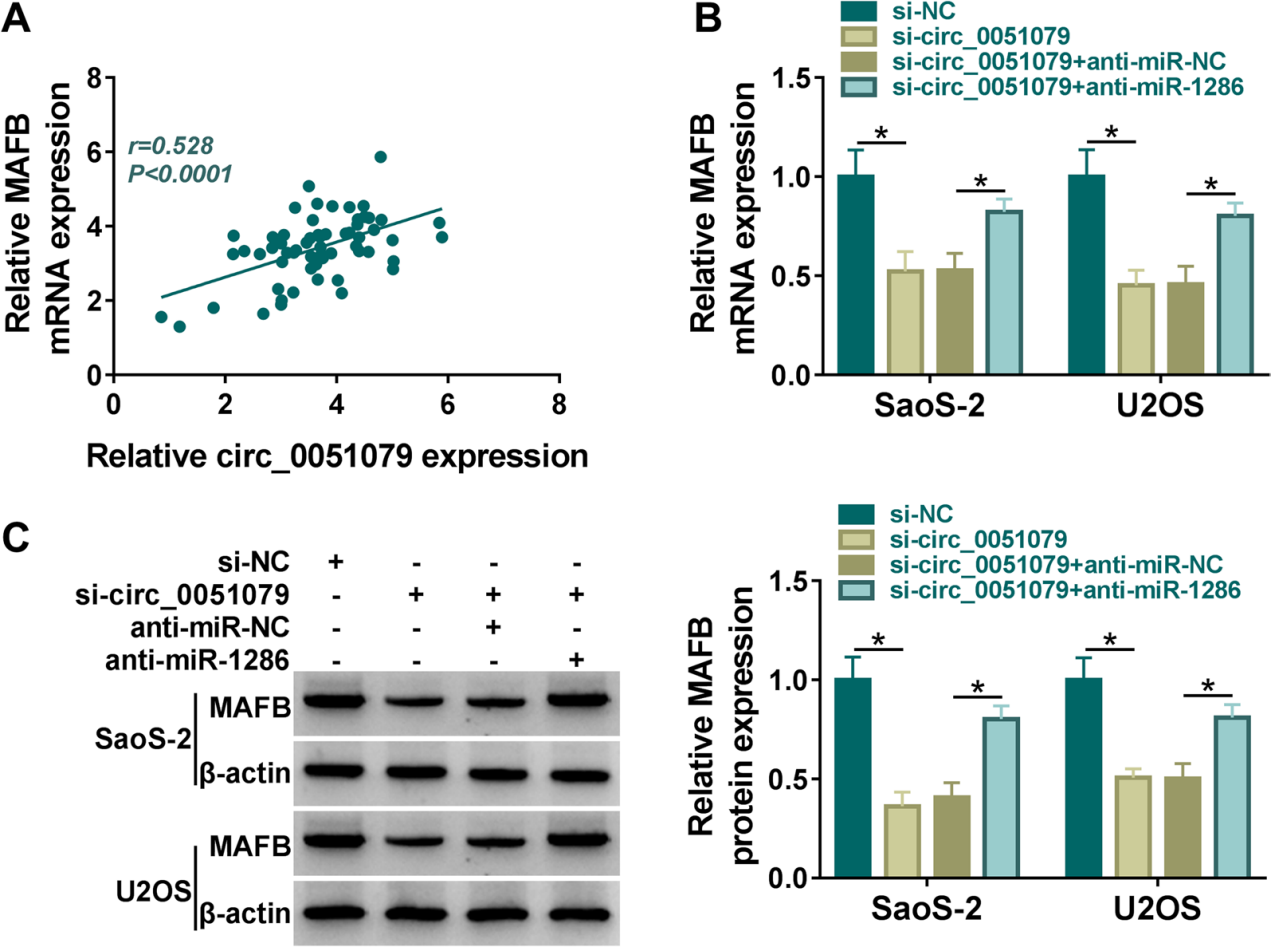
Circ_0051079 interacted with miR-1286 to change MAFB expression. **A** The relation between MAFB and circ_0051079 was analyzed via Pearson’s correlation coefficient. **B**, **C** MAFB mRNA and protein examination was carried out through RT-qPCR and western blot in si-circ_0051079, si-circ_0051079 + anti-miR-1286 and relative control groups. *P < 0.05

Interestingly, there was a positive correlation (*r* = 0.528, *P* < 0.0001) between circ_0051079 and MAFB expression levels in OS tissue samples (Fig. 7A). Moreover, downregulation of cic_0051079 obviously repressed MAFB mRNA/protein, while anti-miR-1286 transfection partly abolished these effects in SaoS-2 and U2OS cells (Fig. 7B, C). It was significant that circ_0051079 regulated MAFB expression via competitively binding to miR-1286.

### Circ_0051079 knockdown reduced OS tumorigenesis in vivo depending on miR-1286/MAFB axis

**Fig. 8:**
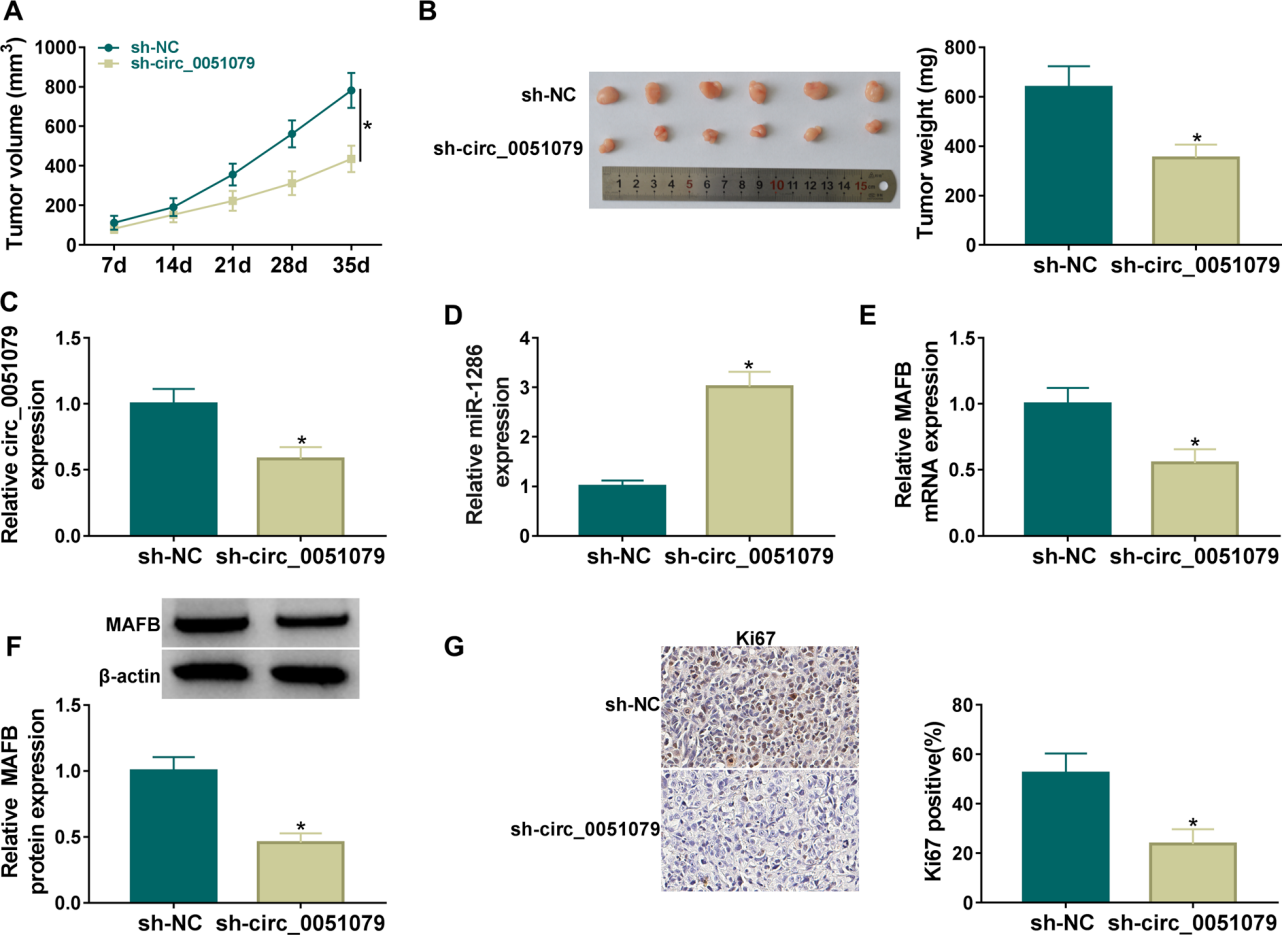
Circ_0051079 knockdown reduced OS tumorigenesis in vivo by depending on miR-1286/MAFB axis. **A**, **B** Tumor volume (**A**) and weight (**B**) of sh-NC and sh-circ_0051079 groups in mice. **C**, **D** RT-qPCR was performed for determination of circ_0051079 (**C**) and miR-1286 (**D**). **E**, **F** MAFB mRNA and protein levels were measured using RT-qPCR and western blot. **G** Ki67 protein level was examined using IHC assay. *P < 0.05

Tumor xenograft assay suggested that tumor volume (Fig. 8A) and weight (Fig. 8B) in mice of sh-circ_0051079 group were reduced relative to those in the sh-NC group. Compared with the sh-NC group, the expression of circ_0051079 was downregulated in tumor tissues of sh-circ_0051079 group (Fig. 8C), and the expression of miR-1286 was upregulated (Fig. 8D). The results of RT-qPCR and western blot demonstrated that circ_0051079 knockdown resulted in inhibition of MAFB mRNA and protein level in mice (Fig. 8E, F). In addition, IHC staining exhibited that Ki67 protein expression was decreased after circ_0051079 silencing (Fig. 8G). Altogether, circ_0051079/miR-1286/MAFB axis regulated OS tumor growth in vivo.

## Discussion

This research unraveled that circ_0051079 might increase MAFB level by competitively interacting with miR-1286, thereby promoting OS cell progression and tumor growth. The tumorigenic features of circ_0051079 were partly associated with the miR-1286/MAFB axis in OS.

CircRNAs have tissue/cell expression specificity, and the up-regulation/down-regulation of circRNA is believed to be related to the malignant cell phenotypes of OS [18]. Yang et al. found significant overexpression of circ_001422 in OS samples and confirmed that silence of circ_001422 repressed metastasis and enhanced apoptosis in OS cells [19]. Furthermore, the upregulation of circ_0032462 might promote OS cell invasion and progression [20]. Additionally, circ_0000190 and circ-ITCH reduced OS cell viability and migration and induce anti-tumor responses [21, 22]. Of note, the present work discovered that circ_0051079 expression is correlated with tumor size and TNM stage. Meanwhile, Kaplan–Meier analysis suggested that the patients in high circ_0051079 level group had a lower survival rate. Apart from that, our cellular data evidenced that circ_0051079 downregulation led to inhibition of proliferation, invasion, migration, and EMT processes. Meanwhile, cell apoptosis was accelerated after the knockdown of circ_0051079. The oncogenic function of circ_0051079 in OS was consistent with the previous report [9].

CircRNA/miRNA networks play a key role in OS development [23]. Sun et al. reported that circ_0001649 blocked proliferation ability of OS cells by targeting different miRNAs, including miR-942, miR-338-5p and miR-647 [24]. Li et al. showed that circ_0001721 facilitated OS cell migration via serving as miR-569 and miR-599 sponges [25]. Circ_0001564 and circ_0086996 promoted cell progression in OS through absorbing miR-29c-3p and miR-125b-5p, respectively [26, 27]. In addition, circRNAs have been involved in the malignant progression of breast cancer and cervical cancer via sponging miR-1286 [28, 29]. Herein, molecular interaction between circ_0051079 and miR-1286 was affirmed in OS. Inhibition of miR-1286 reversed si-circ_0051079-mediated tumor inhibition, suggesting that circ_0051079 contributed to OS cell development via controlling the expression of anti-tumor miR-1286.

For miRNA/mRNA interaction in OS, miR-206 and miR-188-5p suppressed cell progression via targeted degrading Notch3 and CCNT2 [30, 31]. In addition, miR-1284 acted as an expression regulator of HMGB1 to impede the migration and proliferation of OS cells [32]. Liu et al. stated that miR-98-5p inhibited OS progression by decreasing the level of CDC25A [33]. Of note, as a transcription factor, MAFB might be selectively expressed in monocytes/macrophages, which were the precursors of osteoclasts [34]. Meanwhile, previous research suggested that MAFB was obviously decreased during osteoclast differentiation [35]. Furtherly, it has been confirmed that the overexpression of MAFB might promote tumorigenesis in OS [14]. The current results demonstrated that miR-1286 directly targeted MAFB to evoke downregulation of mRNA and protein levels, thus suppressing the OS cell development process.

Moreover, circ_0051079 was identified to regulate MAFB expression via targeting miR-1286 in OS cells. Circ_0008259 was found to inhibit OS progression by upregulation of miR-21-5p-related PDCD4 [8]. Furthermore, hsa_circ_0000006 enhanced OS tumorigenesis through sequestering miR-361-3p and increasing LRIG1 expression [36]. Our animal assay manifested that decreased circ_0051079 repressed tumor growth by modulating miR-1286 and MAFB levels. Therefore, circ_0051079 might affect the occurrence and progression of OS by regulating the miR-1286/MAFB axis in part. Frankly speaking, this study had some shortcomings. For example, although we selected the normal muscle tissues that were more than 5 cm from the cancerous tissues and were used as adjacent noncancerous tissues, it might have limitations. More other clinical samples (e.g., serum samples from normal control and OS patients) need to be performed in the future. Tumor prognosis is a confounding factor because the advanced stage and large tumor size are known as one of the strongest negative prognostic parameters. Therefore, although the high expression of circ_0051079 is correlated with tumor size > 5 cm, advanced TNM stage, and low survival rate, the impact of circ_0051079 on tumor prognostic is confounding in this research.

## Conclusion

In conclusion, our experimental data suggested that circ_0051079 upregulated MAFB expression via targeting miR-1286 to facilitate the malignant development of OS. This study disclosed circ_0051079/miR-1286/MAFB axis as a novel regulatory mechanism for circ_0051079 function in OS. These findings elucidate a new regulatory network that might provide novel insight into the identification of potential biomarkers or therapeutic targets for OS.

## Supplementary Information

**Additional file 1. Fig. S1**: Circ_0051079, miR-1286, and MAFB were linked with the overall survival of patients. (A–C) Kaplan–Meier survival analysis was used to analyze the overall survival of OS patients and circ_0051079, miR-1286, or MAFB.

## Data Availability

Not applicable.

## Abbreviations

OS: Osteosarcoma
MAFB: Musculoaponeurotic fibrosarcoma oncogene homolog B
DAPI: Diamidine phenylindole

## References

[1] Jafari F (2020). Osteosarcoma: a comprehensive review of management and treatment strategies. Ann Diagn Pathol.

[2] Sheng G (2021). Osteosarcoma and metastasis. Front Oncol.

[3] Wang C, Jing J, Cheng L (2018). Emerging roles of non-coding RNAs in the pathogenesis, diagnosis and prognosis of osteosarcoma. Invest New Drugs.

[4] Patop IL, Kadener S (2018). circRNAs in cancer. Curr Opin Genet Dev.

[5] Arnaiz E (2019). CircRNAs and cancer: biomarkers and master regulators. Semin Cancer Biol.

[6] Li Z (2021). An update on the roles of circular RNAs in osteosarcoma. Cell Prolif.

[7] Tu C (2020). Emerging landscape of circular RNAs as biomarkers and pivotal regulators in osteosarcoma. J Cell Physiol.

[8] Guan K (2021). Hsa_circ_0008259 modulates miR-21-5p and PDCD4 expression to restrain osteosarcoma progression. Aging.

[9] Zhang Z, Zhao M, Wang G (2019). Hsa_circ_0051079 functions as an oncogene by regulating miR-26a-5p/TGF-beta1 in osteosarcoma. Cell Biosci.

[10] Inoue J, Inazawa J (2021). Cancer-associated miRNAs and their therapeutic potential. J Hum Genet.

[11] Acunzo M (2015). MicroRNA and cancer—a brief overview. Adv Biol Regul.

[12] Wang J (2019). The role of miRNA in the diagnosis, prognosis, and treatment of osteosarcoma. Cancer Biother Radiopharm.

[13] Yang S, Chen M, Lin C (2019). A novel lncRNA MYOSLID/miR-1286/RAB13 axis plays a critical role in osteosarcoma progression. Cancer Manag Res.

[14] Chen Y (2020). MAFB promotes cancer stemness and tumorigenesis in osteosarcoma through a Sox9-mediated positive feedback loop. Cancer Res.

[15] Livak KJ, Schmittgen TD (2001). Analysis of relative gene expression data using real-time quantitative PCR and the 2(-Delta Delta C(T)) method. Methods.

[16] Duan YR (2021). LncRNA lnc-ISG20 promotes renal fibrosis in diabetic nephropathy by inducing AKT phosphorylation through miR-486-5p/NFAT5. J Cell Mol Med.

[17] Sun J (2021). Down-regulation of SNHG16 alleviates the acute lung injury in sepsis rats through miR-128-3p/HMGB3 axis. BMC Pulm Med.

[18] Soghli N (2020). The regulatory functions of circular RNAs in osteosarcoma. Genomics.

[19] Yang B (2021). Circular RNA circ_001422 promotes the progression and metastasis of osteosarcoma via the miR-195-5p/FGF2/PI3K/Akt axis. J Exp Clin Cancer Res.

[20] Gu R (2020). Circular RNA circ_0032462 enhances osteosarcoma cell progression by promoting KIF3B expression. Technol Cancer Res Treat.

[21] Li S (2020). Extracellular nanovesicles-transmitted circular RNA has_circ_0000190 suppresses osteosarcoma progression. J Cell Mol Med.

[22] Ren C (2019). The circular RNA circ-ITCH acts as a tumour suppressor in osteosarcoma via regulating miR-22. Artif Cells Nanomed Biotechnol.

[23] Huang W (2021). CircRNA-miRNA networks in regulating bone disease. J Cell Physiol.

[24] Sun D, Zhu D (2020). Circular RNA hsa_circ_0001649 suppresses the growth of osteosarcoma cells via sponging multiple miRNAs. Cell Cycle.

[25] Li L (2019). Upregulation of circular RNA circ_0001721 predicts unfavorable prognosis in osteosarcoma and facilitates cell progression via sponging miR-569 and miR-599. Biomed Pharmacother.

[26] Song YZ, Li JF (2018). Circular RNA hsa_circ_0001564 regulates osteosarcoma proliferation and apoptosis by acting miRNA sponge. Biochem Biophys Res Commun.

[27] Luo Z (2020). Circular RNA 0086996 regulates growth and migration of osteosarcoma cells via miR-125b-5p. Pathol Res Pract.

[28] Ni J (2021). Silencing of circHIPK3 sensitizes paclitaxel-resistant breast cancer cells to chemotherapy by regulating HK2 through targeting miR-1286. Cancer Manag Res.

[29] Wang H (2020). Circular RNA circ_PVT1 induces epithelial-mesenchymal transition to promote metastasis of cervical cancer. Aging.

[30] Cai WT (2020). MiRNA-206 suppresses the metastasis of osteosarcoma via targeting Notch3. J Biol Regul Homeost Agents.

[31] Wang F (2020). MiRNA-188-5p alleviates the progression of osteosarcoma via target degrading CCNT2. Eur Rev Med Pharmacol Sci.

[32] Lv S, Guan M (2018). miRNA-1284, a regulator of HMGB1, inhibits cell proliferation and migration in osteosarcoma. Biosci Rep.

[33] Liu X, Cui M (2019). MiRNA-98-5p inhibits the progression of osteosarcoma by regulating cell cycle via targeting CDC25A expression. Eur Rev Med Pharmacol Sci.

[34] Kim K (2007). MafB negatively regulates RANKL-mediated osteoclast differentiation. Blood.

[35] Teti A (2013). Mechanisms of osteoclast-dependent bone formation. Bonekey Rep.

[36] Gao Y (2021). hsa_circ_0000006 induces tumorigenesis through miR-361-3p targeting immunoglobulin-like domains protein 1 (LRIG1) in osteosarcoma. Ann Transl Med.

