# Supplementary figures and images for: Circ_0051079 functions as an oncogenic regulator in osteosarcoma by leading to MAFB expression upregulation by competitively interacting with miR-1286
Source: J Orthop Surg Res. 2022 Sep 24;17:428. doi: 10.1186/s13018-022-03297-w (PMC9509595; doi:10.1186/s13018-022-03297-w)

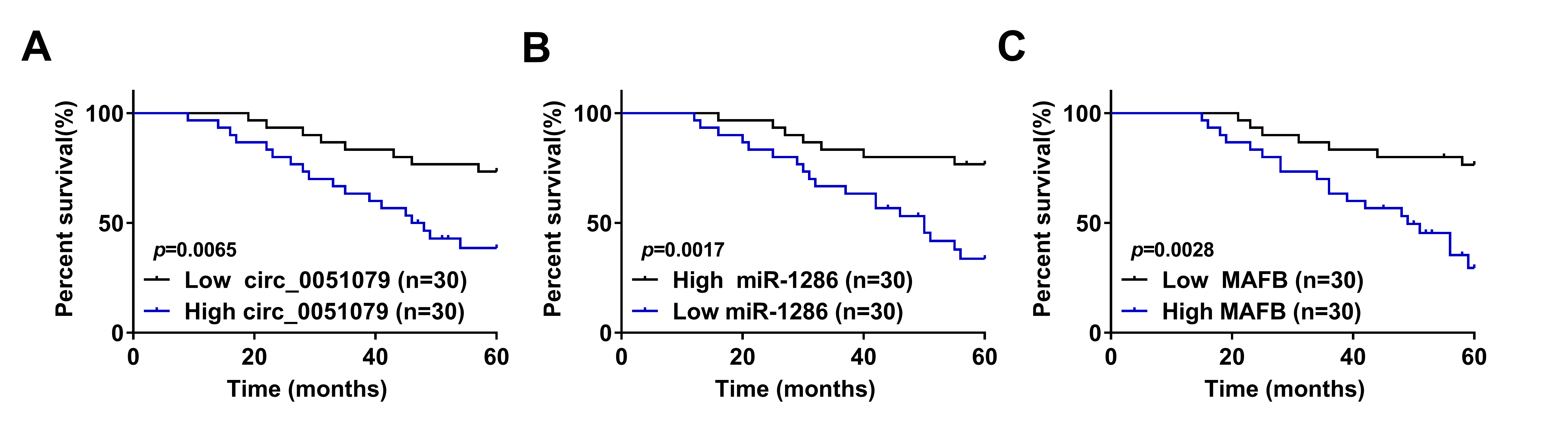

Supplement: Supplementary file 1 — Additional file 1. Fig. S1: Circ_0051079, miR-1286, and MAFB were linked with the overall survival of patients. (A–C) Kaplan–Meier survival analysis was used to analyze the overall survival of OS patients and circ_0051079, miR-1286, or MAFB. [file 13018_2022_3297_MOESM1_ESM.tif]
